# Quo vadis, plasmonic optical tweezers?

**DOI:** 10.1038/s41377-019-0146-x

**Published:** 2019-04-03

**Authors:** Kenneth B. Crozier

**Affiliations:** School of Physics, and Department of Electrical and Electronic Engineering, The University of Melbourne, Melbourne, Victoria 3010 Australia

**Keywords:** Optical manipulation and tweezers, Nanophotonics and plasmonics

## Abstract

Conventional optical tweezers based on traditional optical microscopes are subject to the diffraction limit, making the precise trapping and manipulation of very small particles challenging. Plasmonic optical tweezers can surpass this constraint, but many potential applications would benefit from further enhanced performance and/or expanded functionalities. In this Perspective, we discuss trends in plasmonic tweezers and describe important opportunities presented by its interdisciplinary combination with other techniques in nanoscience. We furthermore highlight several open questions concerning fundamentals that are likely to be important for many potential applications.

## Plasmonics: tweezer opportunities

The progress, challenges, and potential applications for research in plasmonic-assisted optical tweezers are highlighted in this Perspective. Kenneth Crozier from the University of Melbourne in Australia describes how plasmonic nanostructures that confine and enhance electromagnetic fields on a sub-wavelength scale allow optical tweezers to operate beyond the diffraction limit of light and trap nanoscale particles. Potential applications for such plasmonic tweezers include nanoparticle manipulation and sensing, use in “lab-on-a-chip” devices such as flow cytometers for biological studies, or cold atom trapping for quantum information processing tasks. However, before these opportunities can be fully realized it is important that issues such as heating, which occurs due to absorption from the metal structures used in plasmonics, and a more complete understanding of the plasmonic trapping process are properly addressed.

## Introduction

**Fig. 1:**
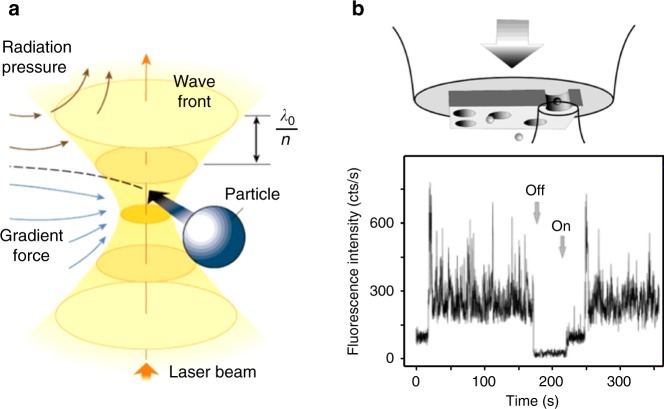
Conventional optical tweezers and plasmonic tweezers (early work). **a** Schematic illustration of conventional optical tweezers. Adapted with permission from Nature: ref. ^1^, Copyright 2003. **b** Fluorescence vs time, showing trapping of a 200-nm latex bead in water by plasmonic tweezers comprising an aperture in Au film. Reprinted with permission from ref. ^2^. Copyright 2004 American Chemical Society

One half of the Nobel Prize in Physics for 2018 was awarded to Arthur Ashkin, “for the optical tweezers and their application to biological systems.” This was truly well-deserved, as optical tweezers (Fig. 1a) have been an important scientific tool in many fields^1^, especially for precise force measurements in biophysics. In this Perspective article, we discuss the use of surface plasmon nanostructures to surpass the limits of conventional optical tweezers, an approach termed “plasmonic tweezers.” Plasmonic tweezers concentrate light into deeply sub-wavelength scales and thus produce narrower and deeper potential wells than conventional tweezers. This capability permits the trapping of nanoparticles at relatively low optical powers with a precision (in position) in keeping with their size. Traditional optical tweezers struggle to achieve this. A small particle near the focused beam of traditional optical tweezers (Fig. 1a) will experience scattering forces (radiation pressure and spin curl force) and the gradient force. The latter is proportional to the gradient of the intensity and is the source of the trapping potential that draws the particle to the laser beam focus. However, the gradient force and trapping potential also vary with the cube of the particle diameter (for a nanosphere), so small particles require high laser powers for stable trapping. Furthermore, the diffraction limit means that the trapping potential well of traditional tweezers can be no narrower than roughly half the wavelength.

An early demonstration of plasmonic tweezers was by Kwak et al., who milled nanoholes in a gold film on a glass substrate and trapped fluorescent latex nanoparticles in water via the enhanced gradient forces produced by the spatially confined fields in the nanoholes (Fig. 1b, ref. ^2^). In other examples of early work, plasmonic tweezers were demonstrated that consisted of pairs of gold particles on glass substrates^3^ and gold nanopillars protruding from a gold film^4^. For the latter, the substrate (silicon) acted as a heat sink, thereby reducing the temperature rise resulting from ohmic losses associated with plasmon excitation. We argue that these and other early works have laid the groundwork for several exciting opportunities in nanoscience for plasmonic tweezers. The structure of this paper is as follows. We begin by discussing these opportunities in the context of the latest advances in plasmonic tweezers. We contend that there are several further challenges that need to be overcome, even in the fundamentals of the trapping mechanism, to realize these possibilities. We conclude by describing our vision of the future potential of plasmonic tweezers.

## Opportunities

**Fig. 2:**
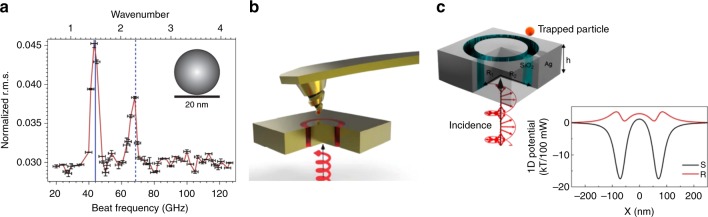
Plasmonic nanotweezers: opportunities as tools for examining the nanoworld. **a** Raman spectrum of polystyrene nanosphere (20 nm), measured using plasmonic nanoaperture tweezer. Reprinted with permission from Nature Photonics: ref. ^6^, Copyright 2014. **b** Schematic illustration of an enantioselective force mapping experiment. Circularly polarized light (CPL) illuminates a coaxial gold nanoaperture plasmonic tweezer, which in turn exerts a force on a chiral atomic force microscope tip that depends on the handedness of the CPL. Reprinted with permission from Nature Nanotechnology: ref. ^8^, Copyright 2017. **c** Simulated trapping potentials for particles (enantiomers, with particle chirality $\kappa = \pm 0.6$) at 20 nm above a plasmonic coaxial aperture illuminated with circularly polarized light (wavelength 751 nm, transmitted power 100 mW). Reprinted with permission from ref. ^9^. Copyright 2016 American Chemical Society

Early works on plasmonic tweezers emphasized the demonstration that small particles could be trapped (e.g., refs. ^2–5^). It was shown that plasmonic tweezers could also sense the presence of the trapped particle (e.g., ref. ^5^). Recent work has expanded the repertoire of sensing modalities of plasmonic nanotweezers to enable them to characterize the trapped object rather than just detect its presence. This broadens the opportunities that plasmonic nanotweezers afford for examining the nanoworld. Wheaton et al., for example, trapped single nanoparticles with a double nanohole (DNH) plasmonic tweezer illuminated by a pair of lasers with slightly different wavelengths. This enabled the determination of the Raman-active acoustic modes of the nanoparticle by measuring fluctuations in the light transmitted through the DNH as a function of the frequency separation of the lasers (Fig. 2a, ref. ^6^). This represents a powerful means to identify unknown nanoparticles (e.g.quantum dots, proteins, and viruses) via their acoustic vibrations. Another example in the theme of examining the nanoworld relates to the analysis of chiral molecules, i.e., those that cannot be superimposed with their mirror images. These forms (“enantiomers”) can interact very differently with other molecules. Enantiomeric purification is thus very important in drug manufacturing, such as in ref. ^7^. Zhao et al. demonstrated coaxial plasmonic aperture nanotweezers which exerted forces that depended on the handedness of the illuminating light and of the object (Fig. 2b, ref. ^8^). The object was a chiral atomic force microscope tip, but one could envision the concept being applied to other nanomaterials, e.g., to sort them by chirality. Such an application was theoretically explored in ref. ^9^, which showed that opposite enantiomers experience different trapping potentials, with one trapped in a deep potential well and the other repelled with a potential barrier (Fig. 2c).

**Fig. 3:**
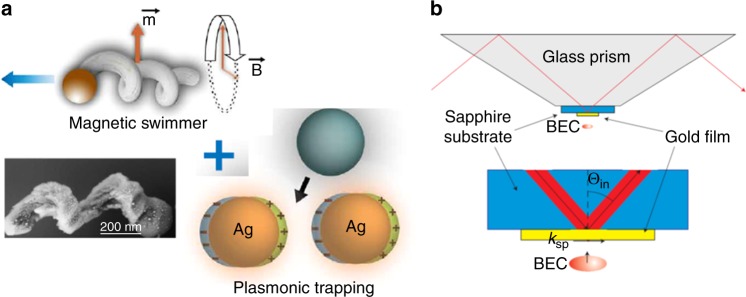
Plasmonic nanotweezers: further opportunities. **a** Example of an opportunity for laboratory-on-a-chip. Integration of a magnetized helical nanostructure (top left) with Ag islands (bottom right) results in a mobile plasmonic nanotweezer. Propulsion of the helical nanostructure can be achieved with a small rotating magnetic field. Bottom left: scanning electron microscopic image, with Ag islands appearing as bright dots. From ref. ^10^. Reprinted with permission from AAAS. **b** Example of an opportunity for integrated devices for ultracold atoms. Schematic of an experiment in which surface plasmons on a gold film are excited by prism coupling and interact with a Bose–Einstein condensate. Reprinted with permission from Nature Photonics: ref. ^11^, Copyright 2011

We next provide glimpses into two more opportunities afforded by plasmonic tweezers. The next opportunity is in the field of laboratory-on-a-chip. Flow cytometers have applications ranging from basic research to the diagnosis of health disorders such as blood cancers. In such systems, cells (or other materials) are suspended in liquid and passed through a detection system to enable measurements such as integrated fluorescence or brightfield/darkfield imaging. These measurements are made on the cells one at a time but at very high speed, thereby enabling rich information to be gleaned about heterogeneous populations. Flow cytometers are thus found in many modern biological laboratories. A current challenge for flow cytometry is the analysis of nanoscale biological materials (e.g., exosomes and viruses). Nanoscience laboratories of the future might contain plasmonic nanotweezer flow cytometers. These would combine plasmonic nanotweezers with lab-on-a-chip microfluidics. The unique sensing capabilities of plasmonic nanotweezers (e.g., refs. ^6,8,9^) are not readily available with traditional optical approaches, and thus nanotweezer flow cytometers could present new opportunities for analyzing heterogeneous populations of nanomaterials. Another potential role in lab-on-a-chip is in addressing the challenge faced by sensor devices based on microfluidic chips that the analytes to be sensed need to diffuse from the center of the channel to its surface (on which the sensors are formed). Mobile plasmonic tweezers that sweep out the three-dimensional volume of the channel could trap nanomaterials and deliver them to sensors (e.g., plasmonic nanotweezers) formed on the surfaces of the channel for precise analysis. Recent work demonstrates this principle. Ghosh and Ghosh demonstrated mobile plasmonic nanotweezers^10^ comprising helical ferromagnetic nanostructures with surfaces decorated by silver nanoparticles. Colloidal beads were trapped by the Ag nanoparticles and then transported to a new location by applying a rotating magnetic field to move the entire helical nanostructure (Fig. 3a).

The third opportunity we suggest for plasmonic tweezers is that of integrated structures for cold atom trapping. There is currently much interest in quantum information networks based on trapped ultracold atoms coupled to nanoscale optical cavities. In some of these demonstrations, the atoms were trapped with optical tweezers in a free-space configuration (i.e., with light focused by a lens)^12^. The use of plasmonic tweezers represents an interesting alternative that offers the possibility of higher levels of integration. Stehle et al. made an important first step in this direction by observing the interaction between Bose–Einstein condensates with the optical near-fields above plasmonic structures (Fig. 3b, ref. ^11^). Mildner et al. recently showed that plasmonic nanostructures could further permit complex trap geometries, such as lattice heterostructures and Fibonacci lattices^13^.

## Challenges

Heating in plasmonic tweezers has long been recognized as a challenge inherent to the technique. In a seminal paper, Novotny et al. theoretically suggested trapping using surface plasmons on a metal tip^14^ but later reported^15^ that trapping experiments were strongly affected by eddy currents “… generated by laser heating of the metal tip.” In pioneering work in 2007, Righini et al. trapped beads using surface plasmons on gold pads^16^. It was noted that heating could produce convection streams that “…may play a significant role in the trapping process…”. In 2010, Ploschner et al.^17^ performed a computational study of trapping with a plasmonic nanoantenna and suggested that the particle localization reported in ref. ^18^ “… may have been due to means other than optical forces…” such as heating. As discussed in the introduction, Wang et al. demonstrated that integrating a heat sink into a plasmonic tweezer drastically reduces the issue of heating^4^. It was also shown that illumination of a gold disk on glass (i.e., without the heat sink) at an intensity sometimes typical of plasmonic tweezers (8 mW/μm^2^) could result in boiling of water^4^. The situation is less problematic for plasmonic nanotweezers based on nanoapertures (in metal films), as the metal film itself facilitates heat dissipation^6^. Xu et al. performed simulations that predicted that a temperature rise of ~6 K would result from illumination of a nanoaperture in a gold film at an intensity of 6.67 mW/μm^2^ (at $\lambda _0 = 1064\;nm$)^19^. This would represent a modest value for many applications but might be too much for some experiments. In such cases, the challenge of heating that accompanies plasmonic tweezers remains, and non-plasmonic approaches can be considered. Xu et al. recently demonstrated an optical nanotweezer based on a dielectric nanoantenna^20^. While the optical forces were smaller than those of many plasmonic designs, heating was substantially less^20^. Lastly, we note that, rather than representing a problem that needs to be overcome, heating can be favorable in some applications. Ndukaife et al., for example, demonstrated that the combination of plasmonic heating and an applied electric field can result in fluid motion that can be employed for particle transport^21^. While considerable progress has been made in addressing the challenge of heating since the early days of plasmonic tweezers, understanding its influence and how to control it remain important questions. This is true for both when it is desired and when it is not.

**Fig. 4:**
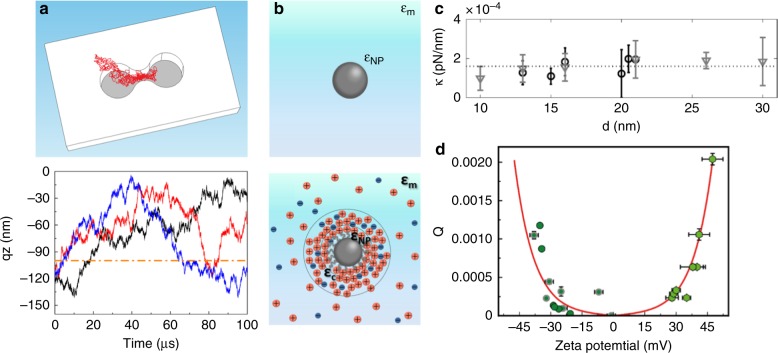
Plasmonic tweezers: challenges **a** Brownian dynamics simulation of a nanoparticle in double nanohole (DNH) plasmonic tweezers. The lower panel shows three simulated nanoparticle trajectories, each starting at the same position $\left( {x_0,y_0,z_0} \right) = (0,0, - 110\;{\mathrm{nm}})$, i.e., centered over DNH and at 10 nm from the gold surface. Reprinted with permission from ref. ^19^. Copyright 2018 American Chemical Society. **b** The optical force on a nanoparticle will be different if the particle is ligand-free (top) or if it contains ligands (bottom). Reprinted with permission from ref. ^22^. Copyright 2018 American Chemical Society. **c** Measured trapping spring constants of quantum dots vs total diameter *d* based on values for the diameter given by the manufacturer (black circles) or by transmission electron microscopy (gray triangles). Reprinted with permission from ref. ^23^. Copyright 2010 American Chemical Society. **d** Measured trapping efficiencies of nanoparticles vs zeta potential. Red line: model. Reprinted with permission from ref. ^22^. Copyright 2018 American Chemical Society

A second challenge facing plasmonic tweezers is the fundamental understanding of the trapping process. One of the reasons that conventional optical tweezers have proved useful for many applications (e.g., ref. ^1^) is the availability of models that can accurately predict the behaviors of particles near a focused beam. Rohrbach, for example, demonstrated very good quantitative agreement between theory and experiment on the trapping behavior of spheres of sub-wavelength diameter^24^. However, such agreement has eluded plasmonic tweezers. This can be understood by considering the Langevin equation for the motion of a particle in an optical trap (e.g., refs. ^19,25^):

1 $$m_{\mathrm{p}}\frac{{{\mathrm{d}}^2\vec r}}{{{\mathrm{d}}t^2}} = \vec F_{\mathrm{D}} + \vec F_{\mathrm{g}} + \vec F_{\mathrm{B}} + \vec F_{{\mathrm{opt}}}$$

where *m*_p_ and $\vec r$ are the mass and position vector of the particle, respectively. The terms $\vec F_{\mathrm{D}}$, $\vec F_{\mathrm{g}}$, $\vec F_{\mathrm{B}}$, and $\vec F_{{\mathrm{opt}}}$ are the drag force, gravity force with buoyancy, Brownian force, and optical force, respectively. We note that Eq. (1) does not explicitly incorporate thermophoresis, except for the case where heating results in fluid flow and thus an additional force on the particle due to Stokes drag. In a conventional optical tweezer, the optical force can be predicted a priori because the fields of the focused beam are known from vector diffraction theory and their interaction with a sphere can be understood by Mie scattering. As the trapping is performed in an (approximately) unbounded medium, the standard expression for the Stokes drag force (proportional to the sphere diameter, sphere velocity, and water viscosity) is applicable^26^. The Brownian force is a random Gaussian process. In conventional optical tweezers, one can furthermore drop the inertial term (left-hand side of Eq. (1)) and the gravity with buoyancy term^26^. All terms of Eq. (1) can thus be predicted a priori for conventional tweezers that trap spherical particles in homogeneous media, provided that the laser power, microscope lens numerical aperture, particle properties (diameter and refractive index), and refractive index of the medium are known. Indeed, Eq. (1) can be Fourier transformed so that the power spectrum of the trapped particle can also be predicted a priori^26^. Why this cannot be readily performed for plasmonic tweezers can be understood by re-examining the force terms of Eq. (1). In plasmonic tweezers, particles are not in an unbounded medium (such as water) but are instead trapped near a surface with a complex morphology for which no compact analytical solution for the drag force $\vec F_D$ exists. An additional term also needs to be included in Eq. (1) to describe the force experienced by the particle when it encounters the surface. The electromagnetic fields have a complex distribution that is associated with the plasmonic nanostructure. The optical force $\vec F_{{\mathrm{opt}}}$ as a function of particle position is thus similarly complex, unlike conventional optical tweezers, for which it can be simply represented by the product of trapping stiffness and particle position. In addition, the presence of the particle will inevitably modify the field distribution. This further complicates the situation. It has been argued that this effect can be beneficial^5^, although this is again complicated by the nature of the particle (e.g., dielectric vs metallic, ref. ^27^). This uncertainty leads to the question of what approach can be taken to predict the trapping process and thus the design of new approaches to plasmonic tweezers that will, for example, allow the opportunities described above to be realized. One is to model Eq. (1) numerically^25^. Xu et al. made a first step in this direction by simulating the Brownian motion of nanoparticles in the vicinity of a DNH (Fig. 4a, ref. ^19^). Trajectories with durations of 100 μs were modeled in three dimensions (e.g., upper panel of Fig. 4a). In Fig. 4a (lower panel), the vertical position (i.e., normal to substrate) is shown as a function of time for three nanoparticle trajectories, showing that the nanoparticles are mostly within the DNH (i.e., $- 100{\mathrm{nm}} < z < 0{\mathrm{nm}}$). While this approach shows promise, there exists considerable scope for further development. This includes predicting particle trajectories over much longer time intervals (to allow comparison to experiment), modeling the drag force accurately, and including other forces such as the particle–surface interaction.

As discussed above, a priori prediction of the behavior of a particle in an optical trap relies on knowledge of the particle’s properties (i.e., diameter and refractive index for a spherical particle). One might expect this to be a trivial matter, but recent work^22,23^ suggests otherwise. We therefore contend that a third challenge facing plasmonic tweezers is how to characterize/model the particle being trapped. This is crucial for future progress, as without it there can be no systematic way of predicting the performance of (and thus evaluating) new types of plasmonic tweezers. One key difficulty is as follows. As noted by Rodríguez-Sevilla et al.^22^, optical tweezers models thus far generally consider the nanoparticle to have a sharp and well-defined interface with the surrounding medium (top panel, Fig. 4b). A more realistic model (lower panel, Fig. 4b) would take the coating layer into consideration. This could comprise coating molecules intentionally added during nanoparticle synthesis or the charge cloud induced in the nanoparticle surroundings, which can be described by the electric double-layer approximation^22^. The need for a realistic model can be understood from the work of Jauffred et al.^23^, who measured the spring constants of (conventional) optical tweezers for the manipulation of colloidal quantum dots. Little connection between the spring constant and nanoparticle diameter were observed (Fig. 4c), even though one would expect an approximately cubic dependence in a conventional nanoparticle model (upper panel, Fig. 4b). Rodríguez-Sevilla et al.^22^ recently made an important step toward resolving this apparent contradiction. They measured the trapping efficiency (*Q*, ref. ^28^) as a function of nanoparticle zeta potential. The latter is indicative of the net charge on a nanoparticle^29^. The measured trapping efficiency vs zeta potential follows a clear trend (Fig. 4d) that is consistent with a model (red line of Fig. 4d) that assumes that the trapping efficiency is proportional to the net charge. One might expect a nanoparticle with a greater net charge to have a larger effective polarizability (for trapping), although (as noted in ref. ^22^) the assumption of proportionality is simplistic, albeit reasonable as a first approximation. We anticipate that future studies testing this model (or a more sophisticated version of it) for plasmonic tweezers (rather than conventional optical tweezers) could be a fruitful contribution to completing our understanding of the physics of the trapping process.

## Future potential

In the (roughly) decade and a half since their introduction, research on plasmonic tweezers has advanced from basic demonstrations to new interdisciplinary applications. Despite this progress, many exciting applications are yet to be fully implemented. We have described some that mainly relate to the field of nanoscience, namely, for examining the nanoworld, laboratory-on-a-chip, and atom optics. Realizing these opportunities will require various challenges to be overcome, such as heating and a fundamental understanding of the physics of the trapping process, even including how to accurately model the nanoparticle (being trapped). In our opinion, the opportunities (and challenges) presented by plasmonic tweezers described in this Perspective article only scratch the surface of what could be possible. It is likely that other possibilities could result from rethinking the common approach to plasmonic tweezers. One example is Brownian motion. Most plasmonic tweezers aim to generate trapping potentials that are as deep as possible to counter the effects of Brownian motion. However, rather than countering it, perhaps Brownian motion could be harnessed by “rectifying” it with a plasmonic nanostructure so that it preferentially occurs in one direction? This would be interesting not only as a fundamental study but also as an alternative means for nanoparticle transport in lab-on-a-chip devices. A demonstration of microparticle transport via this concept using silicon photonic crystals was recently reported^30^. However, no experimental demonstration has been made using plasmonic structures. With many avenues open for investigation and driven by both curiosity and real-world applications, we anticipate that plasmonic tweezers will continue to be actively pursued for some years to come.
